# THE MODERATING ROLE OF SOCIAL SUPPORT IN THE RELATION BETWEEN FUNCTIONAL LIMITATIONS AND DEPRESSIVE SYMPTOMS

**DOI:** 10.1093/geroni/igad104.2738

**Published:** 2023-12-21

**Authors:** Junyub Lim, Giyeon Kim

**Affiliations:** Chung-Ang University, Seoul, Republic of Korea; Chung-Ang University, Seoul, Republic of Korea

## Abstract

**Background and Objectives:**

Prior research has demonstrated that social support from friends and neighbors can effectively reduce depressive symptoms. However, few studies have investigated how the characteristics of these relationships can alleviate depressive symptoms among older adults with functional limitations. Therefore, the present study aims to investigate the moderating effects of characteristics of friends and neighbors relationships on the link between functional limitations and depressive symptoms among Korean older adults.

**Methods:**

The data used for analyses were obtained from the 2020 Survey of Living Conditions and Welfare Needs of Korean Older Persons, a nationally representative survey. The sample included a total of 9,985 individuals who were 65 years of age or older residing in Korea.

**Results:**

Results from the moderator analyses revealed that the link between functional limitations and depressive symptoms was influenced by both the number of close friends and neighbors (b = -0.085, p < .01) and the level of satisfaction with relationships with friends and communities (b = 2.052, p < .05).

**Discussion:**

Satisfaction with relationships with friends and communities may act as a protective factor that mitigates the impact of functional limitations on depressive symptoms. In particular, for older adults with functional limitations, having a larger network of friends and neighbors network is expected to help reduce the risk of depressive symptoms.

